# Polycystic Ovarian Syndrome: Exploring Hypertension and Cardiometabolic Implications

**DOI:** 10.7759/cureus.70958

**Published:** 2024-10-06

**Authors:** Gabriela D Briceño Silva, Karem D Thomas Garcia, Hrachya Ajamyan, Pallavi Shekhawat, Laura C Rodriguez, Ahmad Hammoud, Maria de Jesus Avalos Zapata, Natalia Flores Hernandez, Hilda M Rayon Rayon

**Affiliations:** 1 Obstetrics and Gynecology, Universidad de Oriente, Barcelona, VEN; 2 General Practice, Universidad de Oriente, Barcelona, VEN; 3 General Medicine, Yerevan State Medical University after Mkhitar Heratsi, Yerevan, ARM; 4 Obstetrics and Gynecology, Postgraduate Institute of Medical Sciences and Research (PGIMSR) and Employees' State Insurance (ESI) Model Hospital, Delhi, IND; 5 General Practice, Ilia State University, Tbilisi, GEO; 6 General Practice, Universidad Nacional Autónoma de México (UNAM), Mexico City, MEX

**Keywords:** hyperandrogenism, hypertension, insulin resistance, metabolic syndrome, polycystic ovarian syndrome

## Abstract

Polycystic ovarian syndrome (PCOS) is the most common endocrine disorder affecting women of reproductive age, with significant implications for cardiometabolic health. This review focuses on the relationship between PCOS and hypertension (HTN), an area that remains underexplored despite growing evidence of its importance. PCOS is characterized by hyperandrogenism (HA), ovulatory dysfunction, and polycystic ovarian morphology (PCOM), all of which contribute to a complex metabolic profile that includes insulin resistance (IR), obesity, and dyslipidemia. These factors collectively exacerbate the risk of HTN. Emerging research suggests HA in PCOS may directly influence the renin-angiotensin system (RAS), increasing blood pressure by promoting sodium retention and vascular tone. Additionally, IR, prevalent in both lean and obese women with PCOS, further contributes to HTN by enhancing sympathetic nervous system activity and impairing endothelial function. Despite these associations, the direct link between PCOS and HTN has not been definitively established, warranting further investigation. This review synthesizes current knowledge on the etiology of PCOS and its metabolic consequences, highlighting the need for targeted research to clarify the mechanisms linking PCOS with HTN. Understanding these pathways is crucial for improving the management of PCOS and reducing cardiovascular risks in affected women. By addressing these gaps, this review underscores the importance of considering HTN as a significant comorbidity in PCOS and calls for more comprehensive studies to guide clinical practice.

## Introduction and background

Polycystic ovarian syndrome (PCOS) is a reproductive endocrine disorder that affects seven million women, corresponding to approximately 6-10% of women in the United States [1-3]; it is the most prevalent endocrinopathy in women of reproductive age and is characterized by hyperandrogenism (HA) and either oligo- or anovulation [4]. PCOS is considered a multi-factorial disease with a strong genetic influence, although the exact cause is unknown [5]. 

PCOS has a range of cardiometabolic features, including obesity, intrinsic insulin resistance (IR), gestational diabetes mellitus (GDM), type 2 diabetes mellitus (DM2), hypertension (HTN), dyslipidemia, and subclinical cardiovascular disease (CVD). Extrinsic or obesity-related IR exacerbates the prevalence, severity, and metabolic features of the syndrome [6]. 

Risk factors for PCOS, such as genetics, lifestyle habits, and even environmental factors like exposure to heavy metals, have been recognized [7,8]. Also, the physiological changes of PCOS have been identified, but the relationship between PCOS and HTN remains controversial [9,10]. There is evidence that HTN has a 24% higher prevalence in PCOS patients when compared to healthy females [11]. Metabolic syndrome (MS) associated with IR exists in almost one-third of adolescent and half of adult women with PCOS. Compensatory hyperinsulinemia enhances androgen secretion [12], and it has been postulated that androgen levels may directly control the renin-angiotensin system (RAS) of the proximal renal tubule and increase the reabsorption flow rate, thereby increasing extracellular volume and blood pressure (BP) [9]. Previous research has found that PCOS patients have greater aldosterone levels than age- and body mass index (BMI)-matched controls [11].

This article aims to comprehensively examine the etiology and physiopathology of PCOS, including its metabolic features such as HA, IR, and the RAS and their potential contributions to the development of HTN, as a direct link between PCOS and HTN has not been sufficiently researched. Additionally, it aims to address the considerations when treating PCOS patients, given its status as one of the most prevalent endocrine disorders among women of reproductive age [13].

## Review

Understanding PCOS

PCOS is a diagnosis of exclusion and is a multiorgan disease affecting most endocrine organs, including ovaries, adrenals, pituitary, fat cells, and endocrine pancreas [14]. It is particularly concerning for women of reproductive age [15], as it impacts as many as one in five women in this age group [16]. Studies indicate that Black and non-Hispanic women may have a lower risk of experiencing the full spectrum of symptoms associated with the syndrome compared to Hispanic women, who show a tendency towards a more severe phenotype characterized by a higher likelihood of HA and metabolic imbalances [17]. The manifestations of PCOS are diverse, and up to 50% of patients are normal weight. In most cases, however, the severity of symptoms can be related to abdominal obesity [14].

The diagnostic criteria for PCOS have evolved since its initial description in 1935 by Stein and Leventhal. They first noted enlarged ovaries, obesity, hirsutism, and chronic anovulation in varying degrees [18]. Subsequently, the National Institute of Child Health and Human Development (NICHD) criteria in 1990 stipulated that both chronic anovulation and clinical or biochemical androgen excess were necessary for diagnosis [19]. The Rotterdam criteria (2003) requires at least two of the following: chronic oligo-ovulation or anovulation, clinical or biological HA, and polycystic ovarian morphology (PCOM) on ultrasound [20]. Notably, ultrasonographic evidence of PCOM in a young, healthy woman is not considered a sign of the syndrome, as up to 20-30% of all women may have this feature [21]. Furthermore, as per the recommendation from the international evidence-based guidelines, while using an endovaginal probe with a frequency bandwidth of 8 MHz, a follicle number per ovary of ≥20, or an ovarian volume ≥10 ml on either ovary, ensuring no corpora lutea, cysts, or dominant follicles are present should be the threshold for PCOM on either ovary [22].

In 2006, the Androgen Excess Society (AES) indicated that both clinical and biochemical signs of HA and ovarian dysfunction (oligo-anovulation and PCOM) are required for a diagnosis of PCOS [21]. In 2012, the National Institutes of Health (NIH) recommended maintaining the comprehensive diagnostic criteria of Rotterdam, which encompasses the classic NIH and AES criteria, while identifying different phenotypes based on androgen excess, ovulatory dysfunction, and PCOM [19]. According to the International Evidence-Based Guideline for the Assessment and Management of PCOS, the Rotterdam criteria are the most widely accepted diagnostic criteria [22]. In a 2023 guidelines update, anti-Müllerian hormone (AMH) can now be used instead of ultrasound for diagnosis, offering women a low-cost, convenient diagnostic option without evidence of overdiagnosis with a sensitivity reported at 86% and a specificity of 91% [23]. In adolescents with both HA and ovulatory dysfunction, AMH seems to have low specificity, reported at 45% [23,24].

PCOS has a variety of physiopathological manifestations. Biochemical evidence of HA is seen in 60-80% of women with the syndrome, and about 60% have clinical evidence in the form of hirsutism, acne, and alopecia [25]. The risk of HTN appears to be independent of the BMI. Still, it is exacerbated in patients with obesity, and IR is seen in 75% of lean women and 95% of obese women with the condition [26].

**Figure 1:**
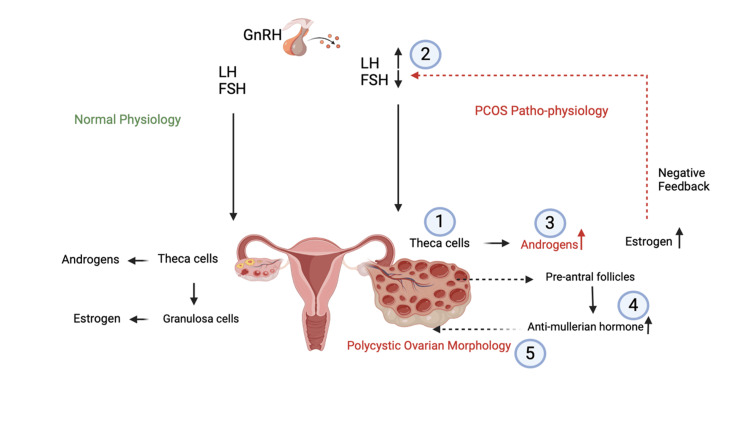
Pathophysiology of PCOS Normal physiological processes alongside the disrupted processes typical of PCOS [27]. Dysfunctional theca cells (1) and an abnormal HPO axis (2) lead to an increased LH/FSH ratio, contributing to ovarian dysfunction and hypersecretion of androgens (3). The excessive androgens, in turn, affect the ovarian microenvironment, causing an increase in anti-Müllerian hormone levels (4) and resulting in polycystic ovarian morphology (5) Figure created by the authors using BioRender and based on information from [27] PCOS: polycystic ovarian syndrome; HPO: hypothalamic-pituitary-ovarian; LH: luteinizing hormone; FSH: follicle-stimulating hormone

Research into the genetic influence of PCOS indicates that the DENNDIA gene, present in ovarian theca cells and adrenal glands, may be overexpressed in theca cells of women with PCOS, particularly its variant 2, which could contribute to excess androgen production [4]. The presence of DENNDIA.V2 in adrenal glands may also account for the simultaneous occurrence of ovarian and adrenal steroidogenesis in nearly 25% of women with PCOS [4]. The overproduction of androgens stems from the dysfunction of theca cells and the hypothalamic-pituitary-ovarian (HPO) axis. Anomalous cellular transduction through negative feedback of estrogen and progesterone leads to irregular gonadotropin-releasing hormone (GnRH) pulsation and gonadotropin secretion, resulting in a high luteinizing hormone (LH) and follicle-stimulating hormone (FSH) ratio that induces ovarian dysfunction and androgen overproduction [27]. The ovarian microenvironment suffers the consequences of this dysfunction due to the elevated levels of AMH secreted by pre-/small antral follicles, leading to the characteristic PCOM [27]. The interactions between PCOS and the HPO axis are better explained in Figure 1. It is not yet clear whether immature follicles, which give rise to the cystic appearance of PCOS, precede HA, hyperinsulinemia, and associated clinical symptoms or vice versa [28].

HA in PCOS

Androgens, a type of steroid hormone, include androstenedione (A4), dihydrotestosterone (DHT), dehydroepiandrosterone (DHEA), testosterone, and dehydroepiandrosterone sulfate (DHEAS). A4, DHEA, and DHEAS are considered precursors of testosterone and DHT. These hormones are crucial for the reproductive hormonal system in women, and their normal synthesis and secretion are essential [29]. Overproduction of androgens, known as HA, is a key clinical feature of PCOS [5]. PCOS is the most prevalent hyperandrogenic disorder, affecting around 80-85% of women with androgen excess [30]. Clinical manifestations of HA may include oligo-/amenorrhea, infertility, acne, seborrhea, hirsutism, and alopecia [5].

Two theories for why HA occurs in PCOS have been proposed. The first theory, the altered gonadotropin secretion theory, postulates that increased GnRH pulse frequency leads to excessive LH and slightly elevated FSH levels. Elevated LH stimulates androgen production from theca cells, while elevated FSH stimulates follicular development and excess estrogen production. The second theory, called the functional ovarian or adrenal HA theory, postulates that HA originates from dysregulated steroidogenesis at the level of the ovary or adrenal gland [29].

There is considerable individual variation in the clinical presentations and biochemistry of HA in women with PCOS. Notably, not all women with PCOS have high levels of testosterone. Some women with PCOS who do not have elevated testosterone levels may still experience acne, hirsutism, and androgenic alopecia due to increased androgens produced by adipose tissue and the adrenal glands rather than from testosterone secretion by the ovaries [31].

In 2012, the NIH consensus panel identified four phenotypes of PCOS (A, B, C, and D). Phenotypes A and B were classified as "classic PCOS," encompassing patients with HA, ovulatory dysfunction, and PCOM under phenotype A, while phenotype B included patients with only HA and ovulatory dysfunction. Phenotype C, termed "ovulatory PCOS," consisted of patients with HA and polycystic ovaries. Finally, phenotype D, labeled "non-hyperandrogenic PCOS," included patients with polycystic and dysfunctional ovaries [11]. These phenotypes are summarized in Table 1.

**Table 1:** Phenotypes of PCOS Table showing the different phenotypes in women with PCOS, according to the National Institutes of Health (2012) [11] Table created by the authors based on information from [11] PCOS: polycystic ovarian syndrome

Phenotypes	Hyperandrogenism	Ovulatory dysfunction	Polycystic ovarian morphology
A	+	+	+
B	+	+	-
C	+	-	+
D	-	+	+

The distorted interactions among endocrine, paracrine, and autocrine factors responsible for follicular maturation may contribute to ovarian dysregulation in PCOS. Intrinsic ovarian factors, such as altered steroidogenesis, and external factors, such as hyperinsulinemia, contribute to excessive ovarian androgen production [24]. Elevated androgens support follicular recruitment while also inducing follicular atresia, ultimately leading to the classic appearance of multi-follicular ovaries, or polycystic ovaries, on transvaginal ultrasonography. However, identifying structures and interpreting ultrasound images is operator-dependent and requires training [28,32]. The classic ovarian phenotype seen in a transvaginal ultrasound is enlarged ovaries with string-of-pearl morphology and theca interstitial hyperplasia. However, this is usually not appropriate in younger girls and women with hymen intact [33]; despite the operator expertise role being crucial, point-of-care ultrasound (POCUS) provides a valuable, non-invasive diagnostic tool in this group of patient settings. Still, correct personnel training is required [32,34].

Clinically, PCOS shows long-standing mild to moderate symptoms of androgen excess, such as hirsutism, acne, alopecia, weight gain, subfertility, or menstrual disturbance. The typical biochemical picture observed is mild to moderate elevations in serum T, A4, and DHEAS. Generally, rapid virilization suggests non-PCOS pathology [33]. 

The precise molecular mechanism connecting androgen dysregulation to HTN remains largely unknown. However, according to experimental models, it is suggested that androgen levels may directly influence the RAS in the proximal renal tubule, leading to an increase in the sodium reabsorption flow rate. This, in turn, could raise extracellular volume and BP [9]. An alternative theory proposes that the hyperandrogenic state PCOS contributes to an intensified cardiometabolic profile, resulting in endothelial dysfunction and elevated BP [12]. Additionally, it is postulated that HA may instigate HTN by boosting the production of angiotensinogen in the kidneys. However, the role of HA in HTN in PCOS remains a topic of debate [9].

Further investigation into the relationship between HA and HTN is crucial, given its frequent and problematic implications in women's health in the short term, during pregnancy, and in the long run for patients diagnosed with PCOS.

The impact of the RAS and endothelin in PCOS

A large body of evidence indicates that sex hormones are responsible for sex differences in BP regulation [35-37]. Before menopause, women are more prone to experiencing low BP compared to men, and estrogen may play a significant role in this [38]. Research shows that increases in BP are more prominent in males, at least until later in life, resulting in males having significantly higher BP than age-matched female counterparts [39]. The impact of estrogen on women's BP, vasculature, and sympathetic nervous system is evident during the menstrual cycle, as BP shows an inverse relationship with circulating estrogen levels [38]. Studies suggest that premenopausal women have a lower risk of CVD compared to men of the same age, likely due to the protective effects of estrogen during a woman's fertile years. However, after menopause, decreasing estrogen levels can lead to a significant increase in the risk of CVD, typically seen about 10 years after menopause [40].

The RAS regulates the body's hemodynamic equilibrium, circulating volume, and electrolyte balance [41]. In the human ovary, all components of the RAS have been identified, and it serves a key role in folliculogenesis and follicular atresia [42]. Prorenin, the inactive renin, is produced by ovarian follicular cells at various stages of oocyte maturation. As the ovarian follicle matures, the concentration of prorenin increases and remains elevated until the end of the luteal phase, coinciding with the start of menstruation, after which it decreases along with progesterone levels. Prorenin concentrations in the reproductive system are generally higher than renin concentrations [43].

Follicular atresia is closely linked to PCOS. In atretic granulosa cells, the most expressed isoform of the angiotensin II (AT2) receptor is AT2R, which has been found to induce apoptosis. In follicles, FSH acts as a mild inhibitor of AT2R expression. During the luteal phase, reduced FSH levels relieve the inhibition of AT2R expression. Consequently, elevated AT2 levels stimulate granulosa cell apoptosis, promoting the atresia of immature follicles [43].

In women diagnosed with PCOS, heightened levels of plasma prorenin and renin are observed, exhibiting a positive correlation with serum androgen levels [44,45]. There is a possibility that androgens may contribute to increased BP by upregulating components of the RAS. As a result, elevated levels of RAS components could potentially indicate hyperandrogenic chronic anovulation [44]. Renin and angiotensin expression can be detected immunohistochemically in the thecal cell layer of healthy women, but it is notably expressed in both granulosa and thecal cells. Thecal cells play a significant role in androgen production in the follicle. Treatment of women with PCOS using an angiotensin-converting enzyme (ACE) inhibitor led to a substantial reduction in HTN and serum testosterone, indicating that RAS blockade may inhibit androgen synthesis [44,45]. An exploration of the relationship between RAS components and IR in PCOS indicates a significant association between PCOS and RAS, possibly linked in part to increased IR in patients with the syndrome [46].

**Figure 2:**
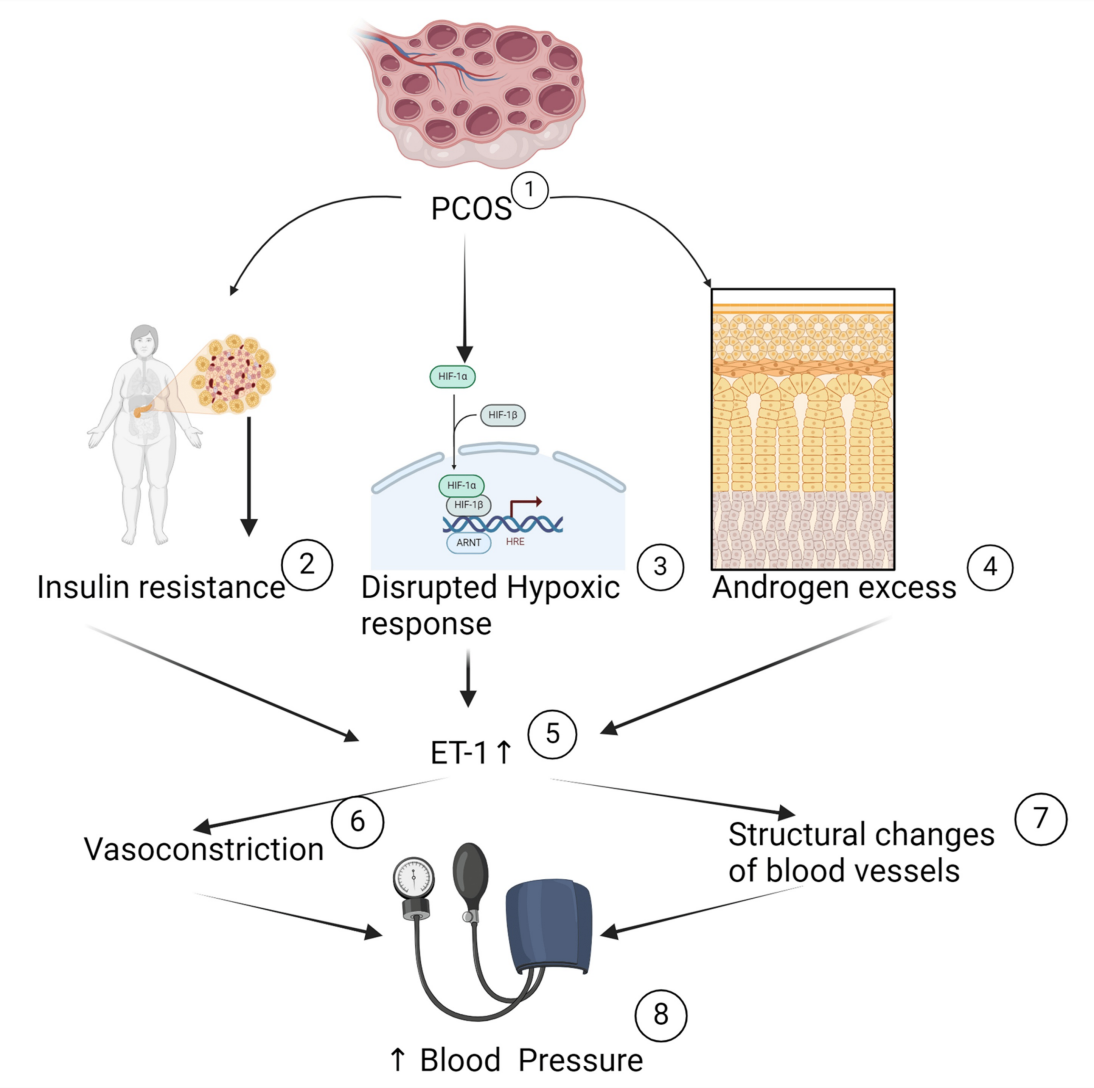
Endothelin's expression in PCOS and its implications in blood pressure PCOS (1) represents an increased risk for insulin resistance (2), disrupted hypoxic response in ovarian granulosa-lutein cells (3), and androgen excess (4). These situations predispose to the increased expression of ET-1 (5). ET-1 is a vasoconstrictor peptide (6) that can also contribute to vascular structural changes, including thickened artery walls and remodeling and lengthening of small arteries (7). These processes are linked to several vascular diseases, including hypertension (8) Figure created by the authors using BioRender and based on information from [47] PCOS: polycystic ovarian syndrome; ET-1: endothelin-1

Endothelins are a family of peptides involved in many physiological processes. Their involvement in PCOS was suggested by the observation that obese and non-obese women with PCOS have higher levels of circulating endothelin-1 (ET-1) compared with controls [47]. The concentration of ET-1 is negatively correlated with follicle diameter. Another endothelin peptide was recently shown to have an essential ovarian function: produced by the granulosa cells around ovulation, endothelin-2 (ET-2) affects follicular rupture and corpus luteum formation. An abnormal endothelin expression pattern in human granulosa cells from PCOS may contribute to PCOS pathogenesis; it may influence the ovulation process and corpus luteum formation in an autocrine/paracrine fashion. ET-1 is known to exert an inhibitory effect on follicular development and luteinization. Therefore, elevated levels of ET-1 in PCOS may interfere with follicular growth and maturation, resulting in the appearance of excessive small follicles and the lack of corpora lutea typical in this syndrome. Furthermore, ET-2, known to promote follicular rupture, is lower in PCOS patients. This, again, may contribute to the ovulation failure observed in these syndrome controls [11,47]. Hypertensive patients do have elevated plasma ET-1 concentrations. It is highly likely that the elevated plasma ET-1 concentrations in hypertensive patients are secondary to HTN and may reflect endothelial cell damage [48]. ET-1's crucial role in HTN is depicted in Figure 2.

The association between metabolic imbalances in PCOS and the risk of HTN

Even though the precise physiological processes are not entirely understood, it is evident that IR is a significant factor in causing HTN in women with PCOS [49]. IR can be present in PCOS patients with average weight, but it becomes more prevalent and severe in cases of obesity, which is an independent risk factor for CVD [50].

Women with PCOS tend to accumulate visceral abdominal fat, leading to chronic low-grade inflammation and elevated inflammatory markers like C-reactive protein (CRP), tumor necrosis factor-α (TNF-α), and interleukin-6 (IL-6), which increase their metabolic risk regardless of BMI [1,5]. In obese women with PCOS, hepatic IR may be prevalent due to intrinsic defects in insulin signaling or receptor activity, decreased insulin clearance caused by high testosterone levels, excess adiposity, and elevated production of free fatty acids or cytokines [50]. When cells become less responsive to insulin, blood sugar regulation is disrupted, leading to compensatory hyperinsulinemia. This condition affects lipid metabolism, protein synthesis, and androgen regulation, contributing to this population's hormonal and metabolic imbalances, possibly contributing to HTN through different pathways [49,51]. The paths through which IR contributes to HTN in PCOS include insulin stimulating the sympathetic nervous system, which increases heart rate and interferes with endothelium-dependent vasodilation. This results in vascular muscle wall hypertrophy and vasoconstriction due to diminished nitric oxide (NO) production, ultimately raising BP. Additionally, compensatory hyperinsulinemia may be linked to imbalances in the autonomic nervous system and increased renal sodium reabsorption due to high aldosterone levels. However, the exact mechanism of this latter effect remains unclear [52-54].

In women with PCOS, there is a notably high prevalence of MS, and the definition of MS includes elevated BP [14]. Studies conducted in the United States have shown that the occurrence of MS in women with PCOS is between 43% and 47%, which is approximately twice as high as in control populations matched for age and BMI [50]. This indicates that PCOS itself, potentially through the promotion of abdominal fat accumulation, elevates the risk of developing MS. Compensatory hyperinsulinemia plays a significant role by sensitizing the ovarian theca layer to augment androgen secretion in response to LH, and it seems to have a similar impact on the adrenal cortex in response to adrenocorticotropic hormone (ACTH). Consequently, the hyperandrogenic state in PCOS worsens the cardiometabolic profile, resulting in endothelial dysfunction and elevated BP [12]. According to the 2023 international PCOS guideline update, BP should be measured by the time of PCOS diagnosis and thereafter annually in all women with the condition [24,55]. Furthermore, young normal-weight women with PCOS have shown significantly higher BP profiles compared to controls, and a higher BP has been associated with greater waist circumference and unfavorable cholesterol, which supports the importance of metabolic screening [55].

Women with PCOS have reported a more atherogenic lipid profile compared to those without the condition [56]. The lipid profile in women with PCOS is characterized by elevated triglycerides, normal or increased total and low-density lipoprotein (LDL) cholesterol levels, and decreased high-density lipoprotein (HDL) cholesterol. This pattern, particularly in overweight women with PCOS, likely reflects the influence of IR, which is associated with increased triglycerides and reduced HDL cholesterol. Moreover, being overweight or obese further worsens the fasting lipid profile in this population, increasing their CVD risk [49].

IR typical of PCOS leads to increased lipolysis from adipose tissue, contributing to dyslipidemia, which can have toxic effects on pancreatic islet cells, inducing apoptosis and increasing the risk of glucose intolerance, chronic hyperglycemia, and eventually DM2 [54]. Dyslipidemia, the most prevalent metabolic abnormality in PCOS, along with endothelial dysfunction, an early sign of subclinical atherosclerosis, contributes to higher systolic and diastolic BP, making HTN a common clinical concern in these patients [12].

Another cardiovascular condition, independent of IR, has been correlated with PCOS. The development of left ventricular hypertrophy (LVH) without diastolic dysfunction in normotensive women with PCOS is significantly higher compared to obese women without the syndrome [57]. This suggests that the hearts of women with PCOS undergo compensatory changes in left ventricular (LV) structure that are not seen in obese women without PCOS. These changes could be an early indicator of future diastolic dysfunction risk in these women. Nonetheless, isolating the exact mechanisms behind the development of LV remodeling in this population is challenging due to the complex and multifaceted nature of PCOS and its associated conditions and the need to rule out other diagnoses [58-60]. It is well known that HTN, as an independent risk factor, can cause LVH. Therefore, further research is needed to determine whether women with PCOS and associated HTN have a higher likelihood of developing LVH with or without diastolic dysfunction compared to women with HTN without PCOS [58-60].

Current management of PCOS and future directions

Management of PCOS focuses mainly on symptom control as well as factoring in the patient's desire for pregnancy. Obesity, IR, and HA are the most frequent presentations of PCOS [61]; therefore, among the most common treatments are antiandrogenic drugs such as combined oral contraceptives (COCP) and insulin sensitizers like metformin [62]. There is a need to individualize the treatment according to the social environment of the patient and the current needs of the era [32].

General recommendations for PCOS patients include losing weight since an improved diet and regular exercise have proven to increase metabolism, improve insulin sensitivity, and help lose weight safely. Furthermore, inactivity has been associated with obesity, HTN, peripheral IR, and dyslipidemia, as it is prevalent among PCOS patients, according to the MS model [63]. There is evidence that adherence to dietary approaches to stop hypertension (DASH) eating patterns for eight weeks among overweight and obese women with PCOS had beneficial effects on weight, BMI, serum triglycerides, very-low-density lipoprotein cholesterol (VLDL-C), insulin, plasma total antioxidant capacity, and total glutathione (GSH) levels compared with the control diet [64]. Weight loss and improved insulin sensitivity were also seen following energy restriction for 12 weeks in overweight women with PCOS [63]. Reducing up to 5% of one's initial weight can help restore regular menstruation and boost the reaction to ovulation and reproductive medications [65]. Even short-term energy restrictions for four weeks in PCOS patients resulted in lower fasting insulin [57]. All these parameters seem to contribute to a more practical approach to improving the metabolic profile in patients with the syndrome [66].

Metformin is a biguanide medication that has been proven to be both safe and effective for DM2 as well as PCOS. Metformin improves insulin sensitivity in peripheral tissues by lowering hepatic glucose production, boosting glucose absorption, and reducing hepatic glucose synthesis. It reduces dyslipidemia by directly reducing hyperinsulinemia or altering the liver's free fatty acid metabolism. Metformin has been demonstrated in several trials to significantly affect dyslipidemia, although it did not affect total cholesterol levels. Metformin is prescribed to women with PCOS at a beginning dose of 500-850 mg per day, which can be raised to 2000 mg per day if tolerated. Moreover, metformin in higher doses can help people lose weight and improve their lipid profiles, especially if they are obese and have PCOS [67]. 

Emerging as a new therapeutic option for PCOS, GLP-1 receptor agonists offer distinct benefits in treating metabolic disorders. To effectively consider a patient with PCOS for GLP-1 analog therapy, screening for MS is essential as the primary indication and intended use should be to treat obesity (with a BMI >30) or a BMI >27, accompanied by concomitant metabolic dysfunctions such as HTN, dyslipidemia, obstructive sleep apnea (OSA), impaired fasting glucose, impaired glucose tolerance, and DM2 [68]. A randomized study incorporating women with PCOS and obesity who were previously treated with metformin reported more significant BMI reductions with daily administration of liraglutide, which also proved to reduce visceral adipose tissue area [69,70]. 

There is strong evidence that COCP can be used in the management of HA [71]. COCP could effectively reduce free androgens, especially in severe cases with adolescents, and reduce testosterone while improving the levels of sex hormone binding globulins (SHBG), which bind to androgens and reduce their bioavailability. Another antiandrogenic therapy used in women with PCOS is spironolactone. This aldosterone antagonist has been used as a potassium-sparing diuretic in the setting of HTN since the 1950s. It was an unintended association that linked its use with improvement in hirsutism in a woman with PCOS undergoing treatment of HTN, and it has since become the most widely used antiandrogen for female pattern hair loss in the United States [72]. One of the most frequent side effects of spironolactone is reduced BP, which could be of great benefit in patients with PCOS [73].

Management of PCOS should be tailored not only to alleviate symptoms but also to prevent the occurrence of long-term complications. COCP and antiandrogens are the standard care to reduce androgen levels and treat symptoms while providing endometrial protection [74]. The overall goals of therapy for women with PCOS include the mitigation of hyperandrogenic symptoms, management of metabolic abnormalities and reduction of risk factors for DM2 and CVD, prevention of endometrial hyperplasia, planning and obtaining a safe pregnancy if desired, and improving general well-being and quality of life [75].

Considering the different PCOS phenotypes and risk profiles for other comorbidities, future studies should target building algorithms or tools facilitating targeted screening for women with PCOS with high metabolic risk. To further address inconsistencies, more robust evidence is needed with more rigorous studies such as systematic reviews and meta-analyses [76]. In the future, metabolic modulation in young women with PCOS, especially before their pregnancy plans, may be an effective solution to prevent the intergenerational transmission of PCOS and its metabolic disorders [77].

## Conclusions

PCOS is a globally prevalent endocrinological disorder primarily affecting women of reproductive age. A range of factors, including HTN, MS, and IR, alongside the RAS and ET-1 expression, seem to influence the development of HTN in PCOS patients. Although controversial, some theories link androgen dysregulation to HTN. One theory suggests that androgen levels may directly control the RAS of the proximal renal tubule and increase the reabsorption flow rate, thereby increasing extracellular volume and BP. Another theory proposed that the hyperandrogenic state of PCOS tends to exacerbate the cardiometabolic profile, causing endothelial dysfunction and elevated BP. Treatments for PCOS mainly include lifestyle changes as well as antiandrogenic therapy. Considering the prevalence of obesity with PCOS and its association with HTN, maintaining a balanced diet and regular exercise has proven to increase metabolism, improve insulin sensitivity, and help lose weight safely, all contributing to reducing BP. Antiandrogenic therapy, such as spironolactone, is also used to help lower BP in PCOS patients, thus contributing to more effectively controlling HTN. Further research should target screening tools for women with PCOS with high metabolic risk. In addition, metabolic modulation in younger PCOS patients seeking to get pregnant could be a solution for the prevention of metabolic disorders associated with PCOS.

## References

[1] Meyer ML, Malek AM, Wild RA, Korytkowski MT, Talbott EO (2012). Carotid artery intima-media thickness in polycystic ovary syndrome: a systematic review and meta-analysis. Hum Reprod Update.

[2] Ajmal N, Khan SZ, Shaikh R (2019). Polycystic ovary syndrome (PCOS) and genetic predisposition: a review article. Eur J Obstet Gynecol Reprod Biol X.

[3] Khan MJ, Ullah A, Basit S (2019). Genetic basis of polycystic ovary syndrome (PCOS): current perspectives. Appl Clin Genet.

[4] Szczesnowicz A, Szeliga A, Niwczyk O, Bala G, Meczekalski B (2023). Do GLP-1 analogs have a place in the treatment of PCOS? New insights and promising therapies. J Clin Med.

[5] Madnani N, Khan K, Chauhan P, Parmar G (2013). Polycystic ovarian syndrome. Indian J Dermatol Venereol Leprol.

[6] Smet ME, McLennan A (2018). Rotterdam criteria, the end. Australas J Ultrasound Med.

[7] Liang C, Zhang Z, Cao Y (2022). Exposure to multiple toxic metals and polycystic ovary syndrome risk: endocrine disrupting effect from As, Pb and Ba. Sci Total Environ.

[8] Ortiz-Garcia NY, Cipriano Ramírez AI, Juarez K, Brand Galindo J, Briceño G, Calderon Martinez E (2023). Maternal exposure to arsenic and its impact on maternal and fetal health: a review. Cureus.

[9] Joham AE, Kakoly NS, Teede HJ, Earnest A (2021). Incidence and predictors of hypertension in a cohort of Australian women with and without polycystic ovary syndrome. J Clin Endocrinol Metab.

[10] Ibrahim AA, Mohammed HS, Elsaid NM, Salim AA, Fathy EG, Hasaneen NM (2023). Risk factors for polycystic ovary syndrome among women of reproductive age in Egypt: a case control study. Afr J Reprod Health.

[11] Sangaraju SL, Yepez D, Grandes XA, Talanki Manjunatha R, Habib S (2022). Cardio-metabolic disease and polycystic ovarian syndrome (PCOS): a narrative review. Cureus.

[12] Macut D, Mladenović V, Bjekić-Macut J (2020). Hypertension in polycystic ovary syndrome: novel insights. Curr Hypertens Rev.

[13] Lo JC, Feigenbaum SL, Yang J, Pressman AR, Selby JV, Go AS (2006). Epidemiology and adverse cardiovascular risk profile of diagnosed polycystic ovary syndrome. J Clin Endocrinol Metab.

[14] Glintborg D (2016). Endocrine and metabolic characteristics in polycystic ovary syndrome. Dan Med J.

[15] Bozdag G, Mumusoglu S, Zengin D, Karabulut E, Yildiz BO (2016). The prevalence and phenotypic features of polycystic ovary syndrome: a systematic review and meta-analysis. Hum Reprod.

[16] Osibogun O, Ogunmoroti O, Michos ED (2020). Polycystic ovary syndrome and cardiometabolic risk: opportunities for cardiovascular disease prevention. Trends Cardiovasc Med.

[17] Engmann L, Jin S, Sun F (2017). Racial and ethnic differences in the polycystic ovary syndrome metabolic phenotype. Am J Obstet Gynecol.

[18] Stein IF, Leventhal ML (1935). Amenorrhea associated with bilateral polycystic ovaries. Am J Obstet Gynecol.

[19] NATIONAL INSTITUTES OF HEALTH (2024). Evidence-based methodology workshop on polycystic ovary syndrome (PCOS). https://prevention.nih.gov/research-priorities/research-needs-and-gaps/pathways-prevention/evidence-based-methodology-workshop-polycystic-ovary-syndrome-pcos.

[20] (2004). Revised 2003 consensus on diagnostic criteria and long-term health risks related to polycystic ovary syndrome (PCOS). Hum Reprod.

[21] Goodarzi MO, Dumesic DA, Chazenbalk G, Azziz R (2011). Polycystic ovary syndrome: etiology, pathogenesis and diagnosis. Nat Rev Endocrinol.

[22] Teede HJ, Misso ML, Costello MF (2018). Recommendations from the international evidence-based guideline for the assessment and management of polycystic ovary syndrome. Hum Reprod.

[23] van der Ham K, Laven JS, Tay CT, Mousa A, Teede H, Louwers YV (2024). Anti-Müllerian hormone as a diagnostic biomarker for polycystic ovary syndrome and polycystic ovarian morphology: a systematic review and meta-analysis. Fertil Steril.

[24] Witchel SF, Oberfield SE, Peña AS (2019). Polycystic ovary syndrome: pathophysiology, presentation, and treatment with emphasis on adolescent girls. J Endocr Soc.

[25] Teede HJ, Tay CT, Laven JJ (2023). Recommendations from the 2023 international evidence-based guideline for the assessment and management of polycystic ovary syndrome. J Clin Endocrinol Metab.

[26] Guan C, Zahid S, Minhas AS, Ouyang P, Vaught A, Baker VL, Michos ED (2022). Polycystic ovary syndrome: a "risk-enhancing" factor for cardiovascular disease. Fertil Steril.

[27] Harada M (2022). Pathophysiology of polycystic ovary syndrome revisited: current understanding and perspectives regarding future research. Reprod Med Biol.

[28] Dason ES, Koshkina O, Chan C, Sobel M (2024). Diagnosis and management of polycystic ovarian syndrome. CMAJ.

[29] Xu Y, Qiao J (2022). Association of insulin resistance and elevated androgen levels with polycystic ovarian syndrome (PCOS): a review of literature. J Healthc Eng.

[30] Yildiz BO, Bolour S, Woods K, Moore A, Azziz R (2010). Visually scoring hirsutism. Hum Reprod Update.

[31] Chen MJ, Yang WS, Yang JH, Chen CL, Ho HN, Yang YS (2007). Relationship between androgen levels and blood pressure in young women with polycystic ovary syndrome. Hypertension.

[32] Calderón-Martínez E, Peña-Carranza R, Sampieri-Cabrera R (2023). Reflections on the design of an ultrasound study program in medical undergraduate. Revista de la Fundación Educación Médica.

[33] Cussen L, McDonnell T, Bennett G, Thompson CJ, Sherlock M, O'Reilly MW (2022). Approach to androgen excess in women: clinical and biochemical insights. Clin Endocrinol (Oxf).

[34] Sampieri-Cabrera R, Calderon-Martinez E (2023). Ultrasonografía didáctica para profesores de pregrado mediante herramientas virtuales [Article in Spanish]. Ultrasonografía didáctica para profesores de pregrado mediante herramientas virtuales [In Spanish].

[35] Song JJ, Ma Z, Wang J, Chen LX, Zhong JC (2020). Gender differences in hypertension. J Cardiovasc Transl Res.

[36] Maranon R, Reckelhoff JF (2013). Sex and gender differences in control of blood pressure. Clin Sci (Lond).

[37] Reckelhoff JF (2018). Sex differences in regulation of blood pressure. Adv Exp Med Biol.

[38] Caroccia B, Seccia TM, Barton M, Rossi GP (2016). Estrogen signaling in the adrenal cortex: implications for blood pressure sex differences. Hypertension.

[39] Colafella KM, Denton KM (2018). Sex-specific differences in hypertension and associated cardiovascular disease. Nat Rev Nephrol.

[40] Yang XP, Reckelhoff JF (2011). Estrogen, hormonal replacement therapy and cardiovascular disease. Curr Opin Nephrol Hypertens.

[41] Ferrari R (2013). RAAS inhibition and mortality in hypertension. Glob Cardiol Sci Pract.

[42] Uncu G, Sözer MC, Develioğlu O, Cengiz C (2002). The role of plasma renin activity in distinguishing patients with polycystic ovary syndrome (PCOS) from oligomenorrheic patients without PCOS. Gynecol Endocrinol.

[43] Pepin É, Dehboneh SS, Raguema N, Esfandarani MT, Lavoie JL (2017). Role of the renin-angiotensin system in healthy and pathological pregnancies. Renin-Angiotensin System - Past, Present and Future.

[44] Reckelhoff JF (2007). Polycystic ovary syndrome: androgens and hypertension. Hypertension.

[45] Palumbo A, Ávila J, Naftolin F (2016). The ovarian renin-angiotensin system (OVRAS): a major factor in ovarian function and disease. Reprod Sci.

[46] Arefi S, Mottaghi S, Sharifi AM (2013). Studying the correlation of renin-angiotensin-system (RAS) components and insulin resistance in polycystic ovary syndrome (PCOs). Gynecol Endocrinol.

[47] Imbar T, Klipper E, Greenfield C, Hurwitz A, Haimov-Kochman R, Meidan R (2012). Altered endothelin expression in granulosa-lutein cells of women with polycystic ovary syndrome. Life Sci.

[48] Xu M, Lu YP, Hasan AA, Hocher B (2017). Plasma ET-1 concentrations are elevated in patients with hypertension - meta-analysis of clinical studies. Kidney Blood Press Res.

[49] Wanderley MD, Pereira LC, Santos CB, Cunha VS, Neves MV (2018). Association between insulin resistance and cardiovascular risk factors in polycystic ovary syndrome patients. Rev Bras Ginecol Obstet.

[50] Baldani DP, Skrgatic L, Ougouag R (2015). Polycystic ovary syndrome: important underrecognised cardiometabolic risk factor in reproductive-age women. Int J Endocrinol.

[51] Lim SS, Norman RJ, Davies MJ, Moran LJ (2013). The effect of obesity on polycystic ovary syndrome: a systematic review and meta-analysis. Obes Rev.

[52] Wu CH, Chiu LT, Chang YJ, Lee CI, Lee MS, Lee TH, Wei JC (2020). Hypertension risk in young women with polycystic ovary syndrome: a nationwide population-based cohort study. Front Med (Lausanne).

[53] Marchesan LB, Spritzer PM (2019). ACC/AHA 2017 definition of high blood pressure: implications for women with polycystic ovary syndrome. Fertil Steril.

[54] Wekker V, van Dammen L, Koning A (2020). Long-term cardiometabolic disease risk in women with PCOS: a systematic review and meta-analysis. Hum Reprod Update.

[55] Mellembakken JR, Mahmoudan A, Mørkrid L (2021). Higher blood pressure in normal weight women with PCOS compared to controls. Endocr Connect.

[56] Wild RA, Rizzo M, Clifton S, Carmina E (2011). Lipid levels in polycystic ovary syndrome: systematic review and meta-analysis. Fertil Steril.

[57] De Jong KA, Berisha F, Naderpoor N (2022). Polycystic ovarian syndrome increases prevalence of concentric hypertrophy in normotensive obese women. PLoS One.

[58] Jacobson JA, Danforth DN, Cowan KH (1995). Ten-year results of a comparison of conservation with mastectomy in the treatment of stage I and II breast cancer. N Engl J Med.

[59] Calderon Martinez E, Ortiz-Garcia NY, Herrera Hernandez DA (2023). Hypertrophic cardiomyopathy diagnosis and treatment in high- and low-income countries: a narrative review. Cureus.

[60] Wang ET, Ku IA, Shah SJ (2012). Polycystic ovary syndrome is associated with higher left ventricular mass index: the CARDIA Women's study. J Clin Endocrinol Metab.

[61] Rojas J, Chávez M, Olivar L (2014). Polycystic ovary syndrome, insulin resistance, and obesity: navigating the pathophysiologic labyrinth. Int J Reprod Med.

[62] Melin J, Forslund M, Alesi S (2024). Metformin and combined oral contraceptive pills in the management of polycystic ovary syndrome: a systematic review and meta-analysis. J Clin Endocrinol Metab.

[63] Dai B, Jiang J (2021). Increased miR-188-3p in ovarian granulosa cells of patients with polycystic ovary syndrome. Comput Math Methods Med.

[64] Asemi Z, Samimi M, Tabassi Z, Shakeri H, Sabihi SS, Esmaillzadeh A (2014). Effects of DASH diet on lipid profiles and biomarkers of oxidative stress in overweight and obese women with polycystic ovary syndrome: a randomized clinical trial. Nutrition.

[65] Day F, Karaderi T, Jones MR (2018). Large-scale genome-wide meta-analysis of polycystic ovary syndrome suggests shared genetic architecture for different diagnosis criteria. PLoS Genet.

[66] Butt MS, Saleem J, Zakar R, Aiman S, Khan MZ, Fischer F (2023). Benefits of physical activity on reproductive health functions among polycystic ovarian syndrome women: a systematic review. BMC Public Health.

[67] Akre S, Sharma K, Chakole S, Wanjari MB (2022). Recent advances in the management of polycystic ovary syndrome: a review article. Cureus.

[68] Cena H, Chiovato L, Nappi RE (2020). Obesity, polycystic ovary syndrome, and infertility: a new avenue for GLP-1 receptor agonists. J Clin Endocrinol Metab.

[69] Jensterle M, Salamun V, Kocjan T, Vrtacnik Bokal E, Janez A (2015). Short term monotherapy with GLP-1 receptor agonist liraglutide or PDE 4 inhibitor roflumilast is superior to metformin in weight loss in obese PCOS women: a pilot randomized study. J Ovarian Res.

[70] Frøssing S, Nylander M, Chabanova E (2018). Effect of liraglutide on ectopic fat in polycystic ovary syndrome: a randomized clinical trial. Diabetes Obes Metab.

[71] Jia S (2023). Combined oral contraceptives (COCs) management on symptoms of polycystic ovary syndrom: a systematic review. Highl Sci Eng Technol.

[72] Bentley-Lewis R, Seely E, Dunaif A (2011). Ovarian hypertension: polycystic ovary syndrome. Endocrinol Metab Clin North Am.

[73] Armanini D, Andrisani A, Bordin L, Sabbadin C (2016). Spironolactone in the treatment of polycystic ovary syndrome. Expert Opin Pharmacother.

[74] Luque-Ramírez M, Nattero-Chávez L, Ortiz Flores AE, Escobar-Morreale HF (2018). Combined oral contraceptives and/or antiandrogens versus insulin sensitizers for polycystic ovary syndrome: a systematic review and meta-analysis. Hum Reprod Update.

[75] Rocha AL, Oliveira FR, Azevedo RC (2019). Recent advances in the understanding and management of polycystic ovary syndrome. F1000Res.

[76] Calderon Martinez E, Flores Valdés JR, Castillo JL (2023). Ten steps to conduct a systematic review. Cureus.

[77] Che Y, Yu J, Li YS, Zhu YC, Tao T (2023). Polycystic ovary syndrome: challenges and possible solutions. J Clin Med.

